# Unraveling Drug Delivery from Cyclodextrin Polymer-Coated Breast Implants: Integrating a Unidirectional Diffusion Mathematical Model with COMSOL Simulations

**DOI:** 10.3390/pharmaceutics16040486

**Published:** 2024-04-02

**Authors:** Jacobo Hernandez-Montelongo, Javiera Salazar-Araya, Elizabeth Mas-Hernández, Douglas Soares Oliveira, Juan Paulo Garcia-Sandoval

**Affiliations:** 1 Department of Physical and Mathematical Sciences, Catholic University of Temuco, Temuco 4813302, Chile; 2 Department of Translational Bioengineering, University of Guadalajara, Guadalajara 44430, Mexico; 3 Department of Mathematics and Statistics, University of La Frontera, Temuco 4811230, Chile; j.salazar11@ufromail.cl; 4 Faculty of Chemistry, Autonomous University of Queretaro, Campus Pedro Escobedo, Queretaro 76700, Mexico; elizabeth.mas@uaq.mx; 5 Department of Mathematical Engineering, University of La Frontera, Temuco 4811230, Chile; 6 Jandaia do Sul Advanced Campus, Federal University of Parana, Jandaia do Sul 86900-000, PR, Brazil; douglas.oliveira@ufpr.br; 7 Department of Chemical Engineering, University of Guadalajara, Guadalajara 44430, Mexico

**Keywords:** breast implants, cyclodextrin polymers, drug delivery, mathematical model, COMSOL multiphysics

## Abstract

Breast cancer ranks among the most commonly diagnosed cancers worldwide and bears the highest mortality rate. As an integral component of cancer treatment, mastectomy entails the complete removal of the affected breast. Typically, breast reconstruction, involving the use of silicone implants (augmentation mammaplasty), is employed to address the aftermath of mastectomy. To mitigate postoperative risks associated with mammaplasty, such as capsular contracture or bacterial infections, the functionalization of breast implants with coatings of cyclodextrin polymers as drug delivery systems represents an excellent alternative. In this context, our work focuses on the application of a mathematical model for simulating drug release from breast implants coated with cyclodextrin polymers. The proposed model considers a unidirectional diffusion process following Fick’s second law, which was solved using the orthogonal collocation method, a numerical technique employed to approximate solutions for ordinary and partial differential equations. We conducted simulations to obtain release profiles for three therapeutic molecules: pirfenidone, used for preventing capsular contracture; rose Bengal, an anticancer agent; and the antimicrobial peptide KR-12. Furthermore, we calculated the diffusion profiles of these drugs through the cyclodextrin polymers, determining parameters related to diffusivity, solute solid–liquid partition coefficients, and the Sherwood number. Finally, integrating these parameters in COMSOL multiphysics simulations, the unidirectional diffusion mathematical model was validated.

## 1. Introduction

According to the Global Cancer Observatory, breast cancer is the most prevalent cancer among women and is associated with the highest number of deaths [1]. Treatment options for breast cancer include chemotherapy, radiotherapy, lumpectomy, quadrantectomy, and mastectomy [2,3]. The response from patients to mastectomy has improved over time, due to factors such as advances in oncology and patient empowerment with control over the treatment process, which have led to favor plastic-reconstructive surgeries like mammaplasty, which involves breast augmentation using implants [4]. This not only addresses physical changes caused by mastectomy but also provides psychological benefits and boosts self-esteem [5].

However, the postoperative risks associated with augmentation mammaplasty in breast cancer patients are considerably higher than in healthy women who undergo breast augmentation for cosmetic reasons [6]. For example, the reported rate of breast implant-associated bacterial infections ranges from 1.1% to 2.5% in healthy patients, while for women undergoing reconstruction after mastectomy, it is between 1% and 35% [7].

As an alternative that would contribute to reduce postoperative risks associated with mammaplasty in breast cancer patients, our group recently functionalized commercial breast implants with coatings of cyclodextrin polymers as drug delivery systems [8]. We crosslinked two types of cyclodextrins, β-cyclodextrin (BCD) and (2-hydroxypropyl)-β-cyclodextrin (HPBCD), with citric acid on smooth and textured implants to control the release of three therapeutic molecules: pirfenidone (PFD), rose Bengal (RB), and the peptide KR-12 (KR-12). PFD is an antifibrotic and anti-inflammatory agent known for its ability to prevent and resolve fibrous tissue formation. It has been successfully used in the prevention and treatment of breast implant capsular contracture [9,10]. RB, on the other hand, is a xanthenic dye with various approved applications, including its use as a toxic agent against different cancer and microbial cell lines [11,12]. As for KR-12, it is a peptide with antimicrobial and antibiofilm properties, which makes it effective in preventing bacterial infections [13,14].

In this regard, in the realm of drug delivery systems, having a robust mathematical tool to predict and analyze the release kinetics of therapeutic substances is indispensable [15]. Among the myriad factors influencing drug release, diffusion stands out as a primary mechanism in cyclodextrin polymer-based delivery systems [16]. While semiempirical models have long been the easiest and most handy for studying release kinetics in such systems, such as Korsmeyer–Peppas or monolithic solution models [17,18], to glean deeper insight into the release kinetics, mathematical models for the specific systems should be developed. In light of this, our study focuses on applying a specialized mathematical model to elucidate the release and diffusion patterns of three key substances—PFD, RB, and KR-12—from cyclodextrin polymer-coated breast implants. Our proposed model hinges on a unidirectional diffusion process guided by Fick’s second law. To tackle this complex problem, we employed the orthogonal collocation method, a numerical technique adept at approximating solutions to both ordinary and partial differential Equations [19,20]. Consequently, the mathematical model provided parameters related to diffusivity, solute solid–liquid partition coefficients, and the Sherwood number.

Moreover, the obtained kinetic parameters were used to perform drug release simulations by COMSOL Multiphysics software v. 6.0. This package offers an intuitive environment for exploring a diverse array of physical phenomena. Its embedded modules span a wide range of physical analyses, including drug delivery [21,22,23], and the governing equations are formulated for resolution through the finite element method [24]. In that sense, COMSOL Multiphysics was used in this work as a tool for the validation of the proposed unidirectional diffusion mathematical model.

## 2. Materials and Methods

### 2.1. Cyclodextrin Polymer Coating on Breast Implants

Smooth and textured breast implants were procured from the Mentor^®^ brand (Santa Barbara, CA, USA). Samples (1 × 1 ${\mathrm{cm}}^{2}$) of the outer shells of the implants were polymerized with cyclodextrins following the protocol outlined by K. Escobar et al. (2023) [8]. In brief, the samples underwent oxidation with oxygen plasma using a radio frequency power of 18 W with a flow of 100 mL/min of $O_{2}$ at a pressure of less than 0.2 mmHg for 15 min using the Harrick Plasma Cleaner model PDC-32G (Ithaca, NY, USA). Following oxidation, the samples were immersed in a 1% *w*/*v* chitosan solution (pH 4) for 15 min. Subsequently, the samples were rinsed with distilled water (pH = 4) and air-dried at room temperature.

Afterward, the samples were submerged in a cyclodextrin solution, choosing between BCD or HPBCD. This solution comprised 10 g of cyclodextrin, 3 g of sodium hypophosphite as a catalyst, and 10 g of citric acid in 100 mL of distilled water (1–3 μmhos/cm); the reactive chemicals were obtained from Sigma-Aldrich^®^ (St. Louis, MO, USA). The immersion lasted for 15 min, after which the samples were air-dried at room temperature. The cyclodextrin/citric acid polymerization was carried out in an oven (model ZFD-A540, Zhicheng, China) at 140 °C for 30 min. Finally, the samples were rinsed with distilled water and ethanol to remove any unpolymerized residues and then dried.

It is important to highlight that chitosan was included in the process because it acts as an electrostatic bond between the oxidized surface and the cyclodextrin polymers (BCD or HPBCD) [17]: oxidized surface (-)/chitosan grafting (+)/cyclodextrin polymer (-).

### 2.2. Physicochemical Characterizations

The morphology of the cyclodextrin polymer-coated samples was examined using a variable pressure scanning electron microscope (VP-SEM, SU-3500 Hitachi; Tokyo, Japan) at an acceleration voltage of 10 kV. The thickness of samples were obtained from the SEM images that were processed using freely available ImageJ 1.52k software.

Roughness measurements were conducted with a Dektak 150 stylus profilometer (Veeco; Plainview, NY, USA), employing a force of 1.0 mg and a scan speed of 17 μm/s.

The samples underwent chemical analysis using Attenuated Total Reflectance Fourier-Transform Infrared Spectroscopy (ATR-FTIR). An FTIR spectrometer equipped with an ATR accessory employing a zinc selenide crystal (CARY 630 FTIR Agilent Technologies; Santa Clara, CA, USA) was utilized over the range of 4000 to 600 ${\mathrm{cm}}^{-1}$ with a resolution of 1 ${\mathrm{cm}}^{-1}$ (NS = 4). The resulting spectra underwent mathematical processing through data smoothing and normalization.

Canva v. 2.5 was the freely available software used for drawing pictures and processing images.

### 2.3. Peptide Synthesis

The antimicrobial peptide KR-12 (H-Lys-Arg-Ile-Val-Gln-Arg-Ile-Lys-Asp-Phe-Leu-Arg-NH_2_) was obtained by solid-phase peptide synthesis using a microwave-assisted peptide synthesizer Liberty Blue (CEM; Matthews, NC, USA). All amino acids used were Fmoc (fluorenylmethoxycarbonyl)-protected at the α-amino acid. The orthogonal protection group for arginine (Arg) was 2,2,4,6,7-pentamethyldihydrobenzofuran-5-sulfonyl (Pbf), while lysine and aspartic acid were tert-butyl-protected (all amino acids sourced from IRIS Biotech; Marktredwitz, Germany). To obtain a C-terminal amide, an unloaded Fmoc-Rink-Amide-resin with a substitution grade of 0.30 mmol/g (INTAVIS Peptide Service GmbH; Tübingen, Germany) was used. The synthesis was conducted on a 0.1 mmol scale in dimethylformamide (DMF) (IRIS Biotech, Germany), with coupling performed using DIC (Diisopropylcarbodiimid)/Oxyma (ethyl cyanohydroxyiminoacetate) (0.5 M/1.0 M, respectively). Deprotection of the Fmoc-group was achieved using a 20% piperidine solution in DMF. Following synthesis, the peptide was cleaved from the resin using a mixture of 95% trifluoroacetic acid (TFA)/2.5% water/2.5% triisopropylsilane. The cleaved peptide was precipitated in ice-cold methyl tert-butyl ether, centrifuged, and the supernatant discarded. The remaining peptide was solubilized in UHQ water and freeze-dried in a lyophilizer (Martin Christ Alpha 1-4; Osterode am Harz, Germany). The obtained peptide was characterized using MALDI-ToFMS (Autoflex Speed, Bruker Daltonik; Bremen, Germany) with α-CHCA (α-cyano-4-hydroxycinnamic acid) as a matrix. The mass of the desired KR12-peptide of 1570.93 g/mol was confirmed, with a targeted purity of ≥95%.

### 2.4. Drug Release Experiments

The samples, both pristine and coated, were loaded with concentrated solutions of PFD (Mw = 185.22 g/mol), RB (Mw = 973.67 g/mol), and KR-12 (Mw = 1570.93 g/mol). PFD and RB were sourced from Sigma-Aldrich^®^ (St. Louis, MO, USA). To obtain the drug release profiles, the loaded samples were placed into vials containing phosphate-buffered saline (PBS) at 37 °C and subjected to agitation on a horizontal shaker (100 rpm) (NB-2005LN Biotek, Winooski, VT, USA). The released molecules in the withdrawn bulk PBS were analyzed at predetermined time intervals by UV-Vis spectrophotometry (Evolution 220 model, Thermo Scientific, Waltham, MA, USA) at 310 nm for PFD [25], 545 nm for RB [17], and 208 nm for KR-12 [26].

**Figure 1:**
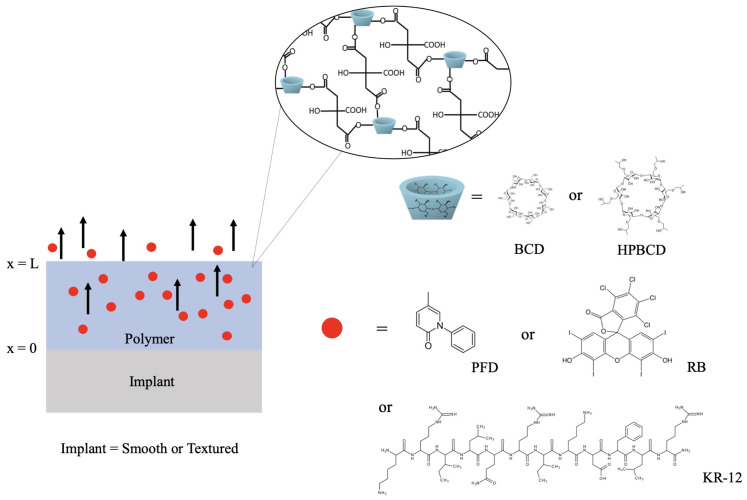
Scheme of the mammary implants (smooth or textured) polymerized with with β-cyclodextrin (BCD) or (2-Hydroxypropyl)-β-cyclodextrin (HPBCD). Samples were loaded with pirfenidone (PFD), rose Bengal (RB), and KR-12 peptide (KR-12).

The release profiles, $C_{l}$*(t)*, were obtained as a mean of triplicate experiments. The experimental procedures are listed in Table 1, and a schematic representation of the performed drug release is illustrated in Figure 1.

## 3. Mathematical Model

### 3.1. Model for a Unidirectional Diffusion Process

The model used in the present study is based on the work of R. Hernandez-Montelongo et al. (2022) [27] and describes the drug delivery process as a unidirectional diffusion, governed by Fick’s second law. The model accounts for convective phenomena from the polymer matrix to the liquid where the drug is delivered, as well as the polymer–liquid drug distribution equilibrium. Here, we present the main equations that describe the model.

Thus, unidirectional diffusion can be described with the following partial differential equation:

(1) $$\frac{\partial C\left(x,t\right)}{\partial t}=D\frac{\partial^{2}C\left(x,t\right)}{\partial x^{2}},\;t>0,\;0<x<L$$

where *C* is drug concentration, *t* is time, *x* is direction along the polymer thickness, *D* is the diffusion coefficient, and *L* is half the length of the sample considered.

It is supposed that the drug load time in the polymer matrix is large enough to have an homogeneous concentration, therefore the initial condition is

(2) $$C\left(x,0\right)=C_{0},$$

where $C_{0}$ is the initial drug concentration in the polymer sample.

On the other hand, due to a symmetric diffusion assumption, in Equation (1), x=0 represents the center of the polymer matrix, while x=L is the surface of the polymer matrix in contact with the liquid where the drug is delivered, therefore the boundary conditions are give by the following equations:

(3) $$\frac{\partial C\left(0,t\right)}{\partial x}=0,$$

(4) $$-D\frac{\partial C\left(L,t\right)}{\partial x}=k_{C}\left[C\left(L,t\right)-\gamma C_{l}\left(t\right)\right],$$

where $k_{C}$ is the mass transfer coefficient in the liquid, γ is the equilibrium coefficient, and $C_{l}$ is the drug concentration in the liquid, which is time dependent due to the drug delivery and initially is considered to be zero, i.e., $C_{l}\left(0\right)=0$.

Moreover, a mass balance in the liquid is used to obtain the time varying drug concentration in the liquid:

(5) $$V_{l}\frac{dC_{l}\left(t\right)}{dt}=Ak_{C}\left[C\left(L,t\right)-\gamma C_{l}\left(t\right)\right],$$

where $V_{l}$ is the liquid volume, and *A* is the area of mass transport.

The initial mass ($m_{0}$) of the drug in the polymer is

(6) $$m_{0}=A\int_{0}^{L}C\left(x,0\right)dx=ALC_{0},$$

assuming the initial concentration in the liquid as zero ($C_{l}\left(0\right)=0$). Therefore, the concentration in the liquid is

(7) $$C_{l}\left(t\right)=\frac{m_{0}-m\left(t\right)}{V_{l}}=A\frac{LC_{0}-\int_{0}^{L}C\left(x,t\right)dx}{V_{l}}.$$

When Equation (7) is substituted in (4), we obtain the following equation:

(8) $$\frac{L}{\mathrm{Sh}}\frac{\partial C\left(L,t\right)}{\partial x}+C\left(L,t\right)=\beta \left[\frac{m_{0}}{AL}-\frac{\int_{0}^{L}C\left(x,t\right)dx}{L}\right],$$

where $\beta =\gamma AL/V_{l}$, and Sh is the Sherwood number defined as $\mathrm{Sh}=\frac{k_{C}}{\left(D/L\right)}$, which is the ratio of the convective mass transfer to the rate of diffusive mass transport. Finally, the drug delivery process can be described by the partial differential Equation (1), the initial condition (2) and the boundary conditions (3) and (8).

### 3.2. Numerical Solution

The model has been solved analytically via a Laplace transformation method [27]. Conversely, in this study, we use numerical methods to solve this model and compare results to the experimental data. The orthogonal collocation method [19,20] was used to numerically approximate the drug concentration within the polymer matrix.

The collocation points are defined by

$$0=x_{0}<x_{1}<x_{2}<\cdots <x_{n}=L,$$

therefore defining the vector of concentrations at the collocation points as

$$\hat{C}\left(t\right)=\left(\begin{array}{c} {\hat{C}}_{0}\left(t\right)\\ {\hat{C}}_{int}\left(t\right)\\ {\hat{C}}_{n}\left(t\right) \end{array}\right)=\left(\begin{array}{c} C\left(x_{0},t\right)\\ C\left(x_{1},t\right)\\ \vdots \\ C\left(x_{n-1},t\right)\\ C\left(x_{n},t\right) \end{array}\right)$$

A system of ordinary differential equations is obtained to calculate concentration inside the domain, for the vector ${\hat{C}}_{int}$:

(9) $$\frac{d{\hat{C}}_{int}\left(t\right)}{dt}=D\left[{\left(M_{2}\right)}_{int,int}{\hat{C}}_{int}+{\left(M_{2}\right)}_{int,0}{\hat{C}}_{0}\left(t\right)+{\left(M_{2}\right)}_{int,n}{\hat{C}}_{n}\left(t\right)\right],\;t>0$$

and an algebraic equation is used for concentration values in the boundaries, ${\hat{C}}_{0}\left(t\right)$, ${\hat{C}}_{n}\left(t\right)$:

(10) $$\begin{array}{c} \left(\begin{array}{c} {\hat{C}}_{0}\left(t\right)\\ {\hat{C}}_{n}\left(t\right) \end{array}\right)\\ ={\left(\begin{array}{cc} {\left(H_{0}M_{1}\right)}_{0} & {\left(H_{0}M_{1}\right)}_{n}\\ {\left(H_{n}+\beta M_{3}\right)}_{0} & {\left(H_{n}+\beta M_{3}\right)}_{n} \end{array}\right)}^{-1}\left[\left(\begin{array}{c} 0\\ \beta M_{3}{\hat{C}}_{0} \end{array}\right)-\left(\begin{array}{c} {\left(H_{0}M_{1}\right)}_{int}\\ {\left(H_{n}+\beta M_{3}\right)}_{int} \end{array}\right){\hat{C}}_{int}\left(t\right)\right], \end{array}$$

where $M_{1}$ is the matrix for the first derivative of the function, $M_{2}$ is the matrix for the second derivative, $M_{3}$ contains values for the integral, $H_{0}$ is defined for the first node, and $H_{n}$ is defined for the last node in the orthogonal collocation method solution. The detailed procedure to obtain Equations (9) and (10) is described in Appendix A.

### 3.3. COMSOL Multiphysics Simulations

To validate the mathematical model, finite element simulations were conducted using the Transport of Diluted Species module in COMSOL Multiphysics software. The simulation involved a two-dimensional representation of silicone, polymer, and PBS. Given that the polymer thickness, *L* (on the order of μm), is significantly smaller than the sample width (on the order of cm), the drug flow near the polymer can be considered perpendicular to the surface. Consequently, the use of a two-dimensional model, instead of a three-dimensional one, does not imply a loss of generality. For the same reason, the chosen width of the model also does not influence the results and was set at 50 μm. It was assumed that there is no drug flow from the polymer to the silicone; thus, the silicone acts solely as a barrier to flow. Therefore, its thickness is also considered non-essential and was set at 20 μm. *L* was chosen based on experimental values, that is, 100 μm for textured implants and 14 μm and 20 μm for smooth implants of BCD and HPBCD, respectively. The thickness of the PBS was chosen to be 100 times *L*. The diffusion coefficient used in the PBS was 10^−8^ m^2^/s, a relatively high value chosen to maintain uniform drug concentration in the PBS, as is experimentally expected due to the sample shaker. The drug diffusion coefficient within the polymer, *D*, and the value of β were derived from the mathematical model. The initial drug concentration in the polymer (*C*_0_) was calculated using the maximum amount of drug released (*A*) as presented in the paper by K. Escobar et al. (2023) [8] and dividing it by L and the molar mass of the drug (*M*), i.e., *C*_0_ = A/LM. The drug release values were obtained from the variation in drug concentration in the polymer matrix.

## 4. Results and Discussion

**Figure 2:**
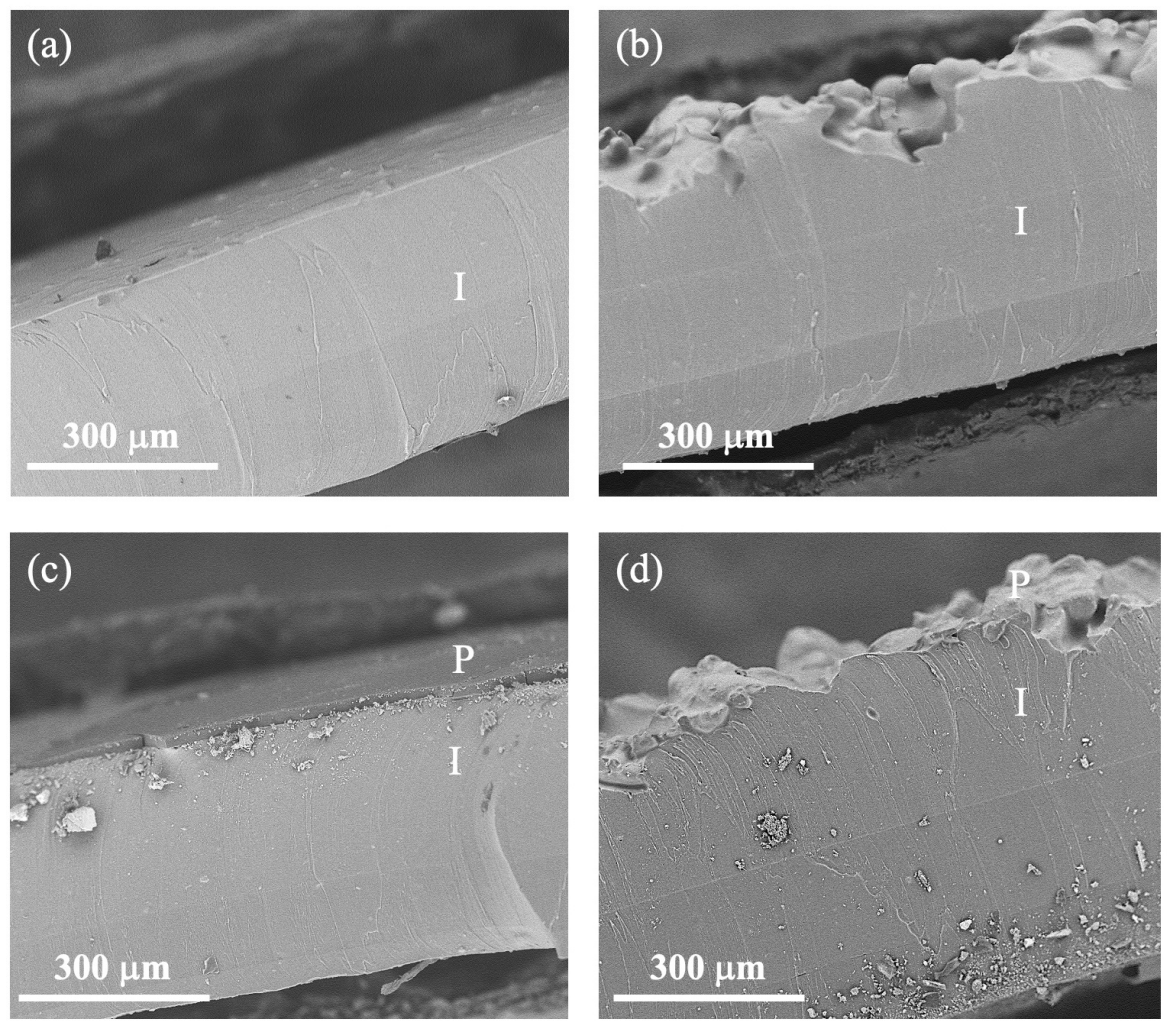
Cross-sectional view of SEM images: (**a**) smooth implant, (**b**) textured implant, (**c**) smooth implant polymerized with HPBCD, and (**d**) textured implant polymerized with HPBCD. P = polymer and I = implant.

Figure 2 displays the cross-sectional scanning electron microscope (SEM) images of the pristine implants, both smooth (Figure 2a) and textured (Figure 2b). The smooth samples exhibited a completely flat surface with a membrane thickness of approximately 380 μm. In contrast, the textured samples featured a thicker membrane, measuring around 520 μm in thickness, and presented valleys with depths ranging from 100 to 150 μm. Figure 5 in the study by K. Escobar et al. (2023) [8] showcases additional perspectives of these substrates.

In Figure 2, the functionalized samples with HPBCD polymer are depicted, including both smooth and textured implants (Figure 2c and Figure 2d, respectively). The resulting coating thickness for the smooth implants was approximately 20 μm, whereas for the textured implants, it measured about 100 μm. The increased thickness in the textured samples can be attributed to the elevated roughness of the surface, leading to polymer accumulation in the bottom of the valleys and adherence to the irregular surface cavities. However, the coating can still be considered a thin film, similar to that of the smooth samples. Regarding the functionalized samples with a BCD polymer, the thickness amounted to around 14 μm for smooth implants and 100 μm for textured implants.

**Figure 3:**
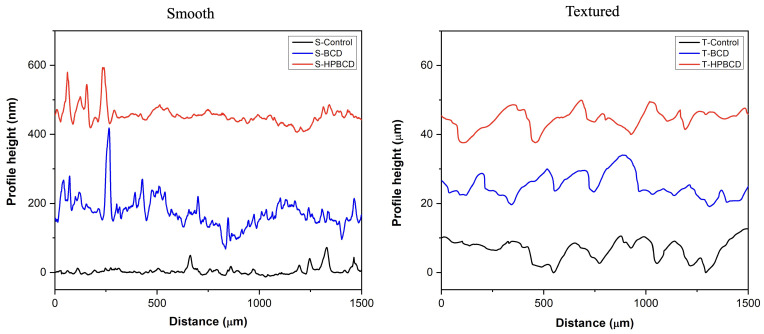
Profile height of smooth and textured implants.

On the other hand, the profile height of the samples has been plotted to better visualize their topography (Figure 3). The valleys and peaks of smooth sample surfaces are in the range of nanometers, while textured samples are in the range of micrometers. Accordingly, the roughness of the smooth surface samples increased from 60 ± 40 nm (control) to 350 ± 50 nm and 300 ± 40 nm for the samples coated with BCD and HPBCD polymers, respectively. For the textured samples, the roughness increased from 12.5 ± 5 μm (control) to 25 ± 10 μm and 20 ± 10 μm for the samples polymerized with BCD and HPBCD, respectively.

**Figure 4:**
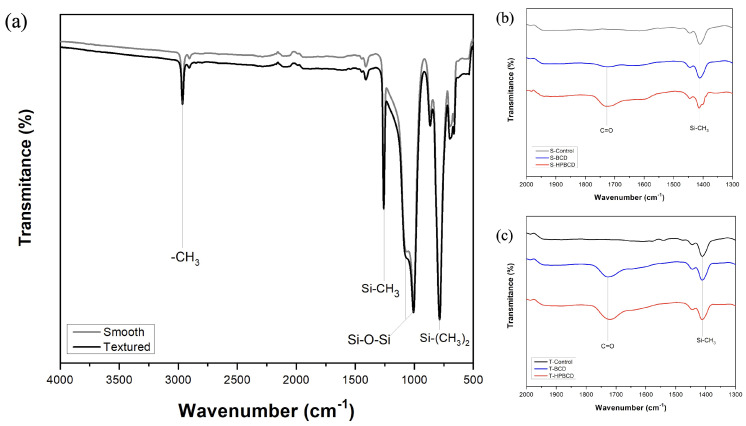
FTIR spectra of samples: (**a**) smooth and textured controls, (**b**) smooth implants coated with BCD and HPBCD polymers, and (**c**) textured implants coated with BCD and HPBCD polymers.

Figure 4 presents the results of chemical analysis conducted on the samples, performed using ATR-FTIR to directly identify the polymer coating. The vibrations of fingerprint functional groups from both types of silicone implants were detected (Figure 4a) [28]: the peak at 2960 ${\mathrm{cm}}^{-1}$ corresponds to the stretching vibration of methyl (-CH_3_). The stronger absorption peaks at 1260 ${\mathrm{cm}}^{-1}$, 1100–1000 ${\mathrm{cm}}^{-1}$, and 790 ${\mathrm{cm}}^{-1}$ are attributed to Si-CH_3_, Si-O-Si, and Si-(CH_3_)_2_ bonds, respectively. Moreover, in Figure 4b,c, another characteristic peak of silicone rubber was identified, with a weak absorption peak at 1410 ${\mathrm{cm}}^{-1}$ corresponding to the asymmetric Si-CH_3_ stretching. Additionally, in the same figures, the characteristic functional group of cyclodextrin polymers was detected in both BCD and HPBCD coatings [29]: the C=O stretching (1730 ${\mathrm{cm}}^{-1}$) of ester groups resulting from esterification and the residual carboxylic acids of the cross-linking agent.

**Figure 5:**
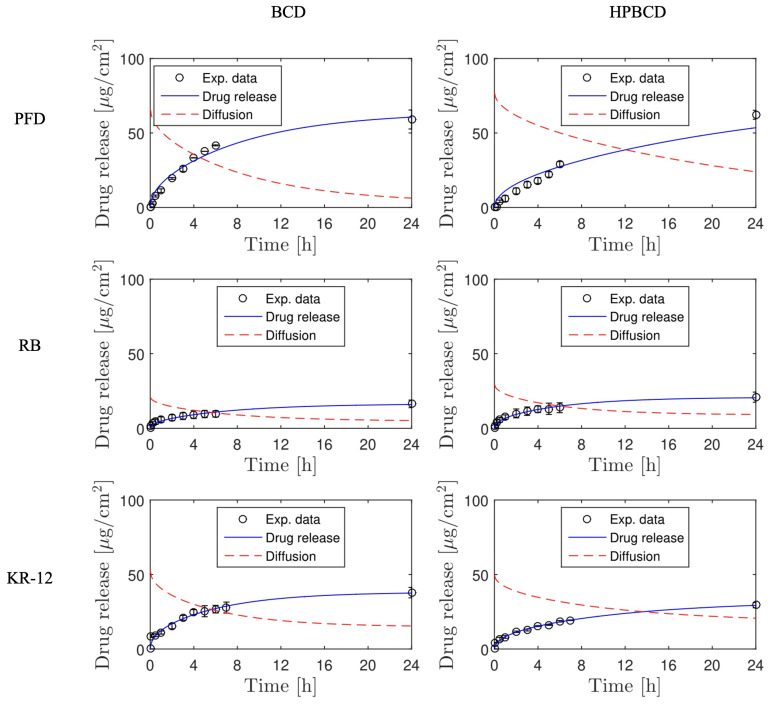
Drug release and diffusion profiles simulated for experiments using smooth implants.

**Figure 6:**
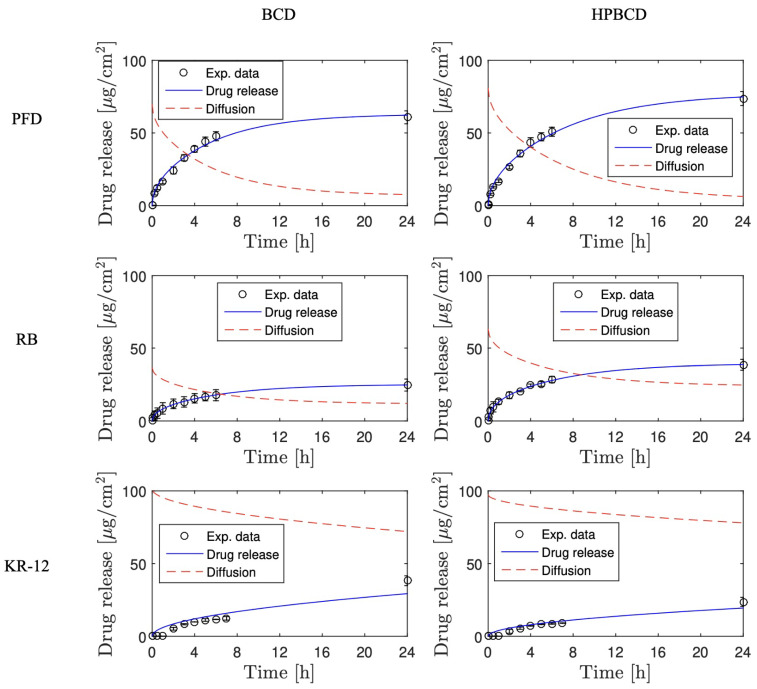
Drug release and diffusion profiles simulated for experiments using textured implants.

The simulation of release profiles for PFD, RB, and KR-12 molecules from polymerized samples with BCD and HPBCD is presented in Figure 5 for smooth implants and Figure 6 for textured implants. Drug release profiles were simulated using experimental data and were well adjusted with the mathematical model. Additionally, the diffusion profiles of drugs through the cyclodextrin polymers are also depicted in the same figures. These simulations were derived directly from the mathematical model due to the absence of experimental data for this particular phenomenon. These results are of significant value, considering that conducting an experimental methodology to obtain diffusion profiles in thin films, especially for micron-level thickness, can be notably costly.

Interestingly, for both smooth and textured implants, the release of 50% of the drugs (PFD, RB, and KR-12) occurred faster in BCD coatings compared to HPBCD coatings. This trend is observed in the intersection of drug release profile simulations and diffusion profile simulations. For instance, in the case of PFD release from smooth implants, the BCD-coated sample reached 50% release around 4 h, whereas the HPBCD-coated sample reached it at 12 h. This difference can be attributed to the higher degree of polymerization exhibited by the HPBCD molecule compared to native BCD [30]. The hydroxypropyl groups of HPBCD enhance the reactivity of the –OH groups present in cyclodextrin, facilitating esterification reactions with the cross-linking agent. This results in a denser three-dimensional polymer matrix, which releases the drugs at a slower rate.

However, in the case of KR-12 release from textured implants, there is no intersection between the drug release profile simulation and the diffusion profile simulation, indicating that the 50% release threshold was not achieved within the first 24 h. This observation suggests a strong interaction between the large molecules of the antimicrobial peptide (Mw = 1570.93 g/mol) and the thick cyclodextrin polymer coatings on textured samples (100 μm), regardless of whether it is BCD or HPBCD.

Furthermore, we extracted three crucial parameters from the model, as detailed in Section 3.1: D, β and Sh. Particularly noteworthy is the Sh value, which was notably high, approximately $\left(\mathrm{Sh}\approx 10^{5}\right)$. This Sh value serves as confirmation that diffusion primarily governs the transport at the interface under the given experimental conditions. Moreover, this outcome is in alignment with previous findings as reported by R. Hernandez-Montelongo et al. (2022) [27] for this type of systems.

**Figure 7:**
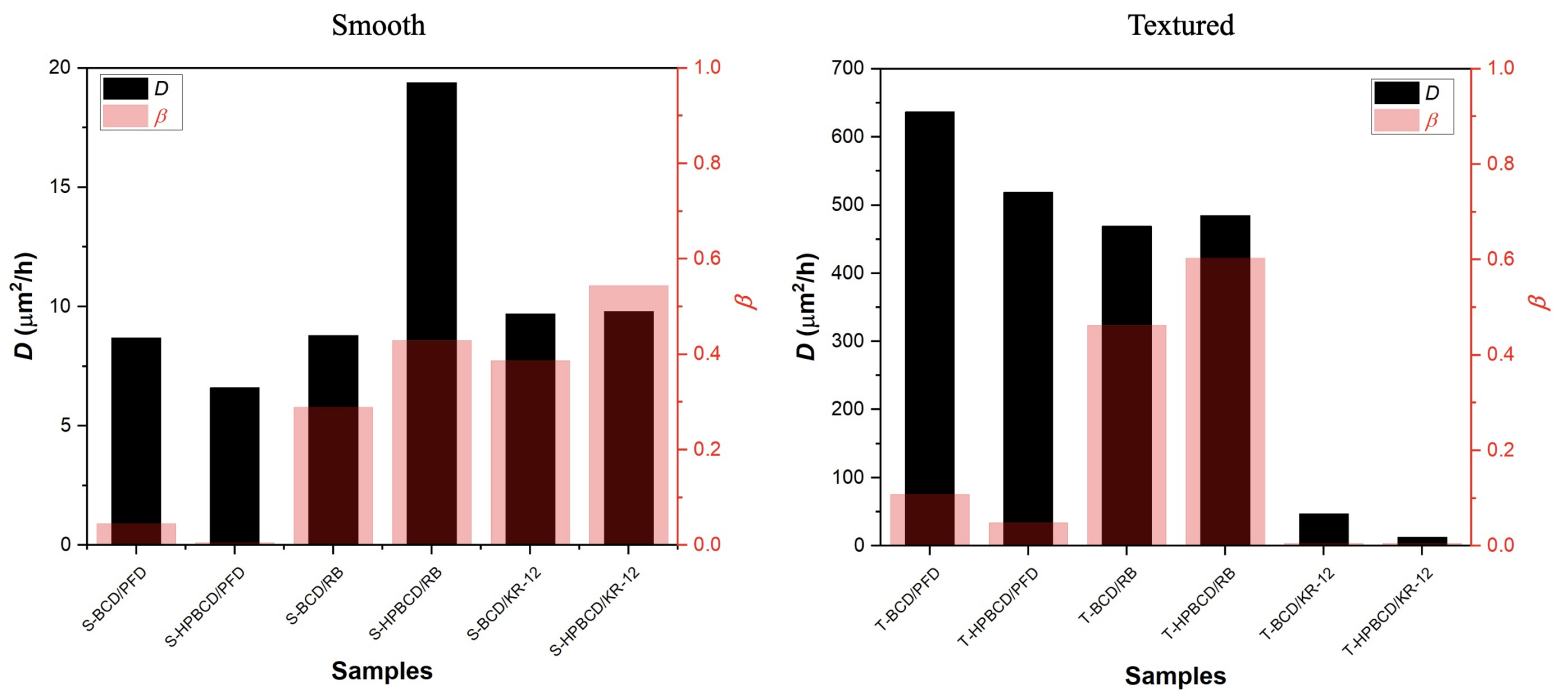
Diffusion coefficient and parameter β for drug release simulations for smooth and textured implants.

The calculated values of *D* and β for both types of implants, smooth and textured, are graphically represented in Figure 7. In the case of smooth samples, the diffusivity values ranged from 6.6 to 19.4 μm^2^/h, and no discernible correlation with the molecular weight of the drugs was observed. Conversely, for textured samples, the *D* values displayed a broader range from 13 to 637 μm^2^/h, exhibiting an inverse relationship with the molecular weight of the drugs, as previously reported by A. Bogdan et al. (2011) [31]. Specifically, lower molecular weight drugs demonstrated higher diffusivity, while higher molecular weight drugs exhibited lower diffusivity. The difference in diffusivity can be attributed to the characteristics of the implants. For smooth implants, the thin coating on the upper substrate surface allowed for the rapid adsorption of the PBS solution, facilitating unrestricted drug movement and diffusion [32,33,34]. Conversely, the textured samples, with their thick and irregular polymer coating, adsorbed the PBS solution at a slower rate, thus favoring mass diffusion, in accordance with the molecular weight of the released drugs.

Moreover, in the same Figure 7, values for β are also reported. As a reminder, β is defined as $\beta =\gamma AL/V_{l}$, and is reliant on γ, which represents the drug solid–liquid partition coefficient associated with the equilibrium. This signifies that higher β values correspond to more of the drug being retained within the polymer matrix once equilibrium is attained. It is essential to emphasize that for smooth implants, this parameter was directly correlated with the molecular weights of the drugs: higher molecular weight drugs exhibited higher β values. This trend is reasonable because larger molecules engage in more extensive chemical and physical interactions with the polymer, leading to their entrapment within its three-dimensional matrix. In the case of textured samples, a similar pattern was observed, but only for the PFD and RB drugs. However, when considering samples with released KR-12, their β values tended toward zero. This observation suggests that equilibrium had not been attained within the first 24 h of release. KR-12 is a large molecule with multiple functional groups [26], resulting in slow diffusion through the thick coatings of T-BCD and T-HPBCD samples. This slow diffusion may be attributed to physisorption interactions, which prevent equilibrium from being reached within the given timeframe [35].

Recently, K. Escobar et al. (2023) [8] obtained diffusion coefficient (D) values for these systems using the semi-empirical “monolithic solution” model. This model is applied when drugs are molecularly dispersed in the matrix former or when the drug rapidly dissolves upon water penetration into the system [18]. The authors adapted the model for slab geometry and the early time of release approximation ($M_{t}$/$M_{\infty }$≤0.6). Although their results fall within the same range as our work, they were notably lower for both cases, ranging from 1.3 to 3.5 μm^2^/h for smooth samples and from 4 to 132 μm^2^/h for textured samples. This discrepancy primarily stems from the “monolithic solution” model not accounting for the drug solid–liquid partition coefficient.

**Figure 8:**
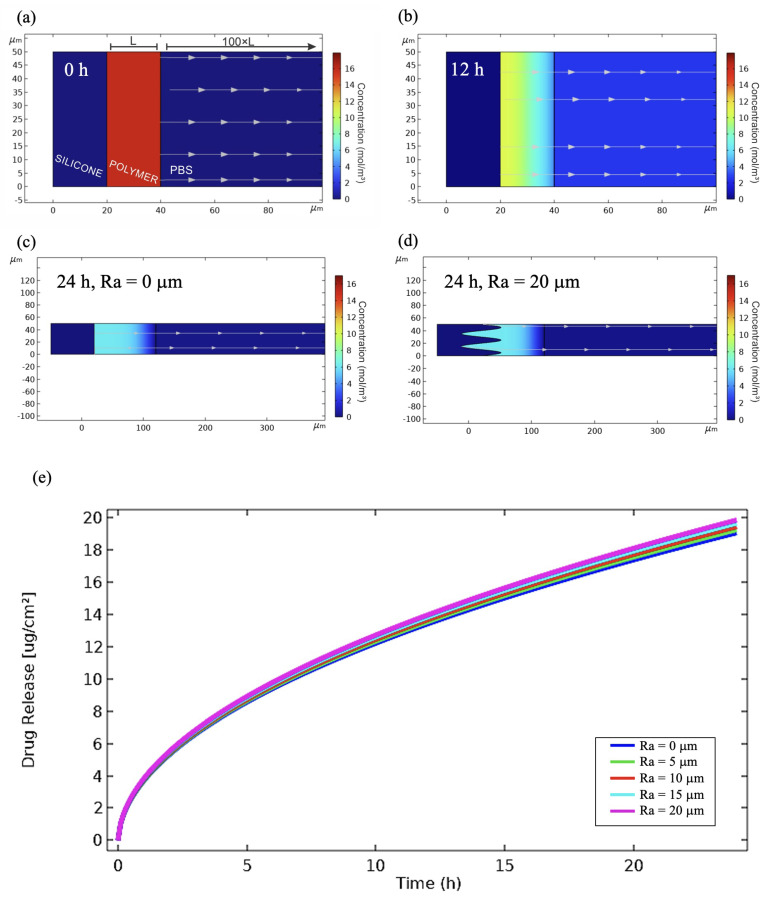
Drug delivery simulations performed by COMSOL Multiphysics: (**a**) S-HPBCD/KR-12 sample at t = 0, (**b**) S-HPBCD/KR-12 sample at t = 12 h, (**c**) T-HPBCD/KR-12 sample with Ra = 0 μm at t = 24 h, (**d**) T-HPBCD/KR-12 sample with Ra = 20 μm at t = 24 h, and (**e**) release profiles of T-HPBCD/KR-12 with different Ra values.

In order to validate the mathematical model, COMSOL Multiphysics simulations were performed using the previously obtained kinetic parameters D and β. Figure 8 shows the two-dimensional COMSOL Multiphysics design used for simulations. As a representative example, Figure 8a presents the case of S-HPBCD/KR-12 at time 0 h and Figure 8b at 12 h of release. The evolution of the drug delivery is observed in the increase in concentration drug in PBS medium and its decrease in the polymer matrix. Moreover, due to the versatility of COMSOL Multiphysics in terms of geometry design, the roughness of the textured samples was modified from an arithmetic average roughness (Ra) of 0 μm to 20 μm (Figure 8c and Figure 8d, respectively), but maintaining the polymer coating as a thin film, similar to that of the smooth samples, just as was considered in the mathematical model. Results show that this assumption was correct because profiles were similar for different cases of Ra with no strong impact on the release profiles of drug (Figure 8e). These values of roughness are in the range reported for this type of textured implants [36].

**Figure 9:**
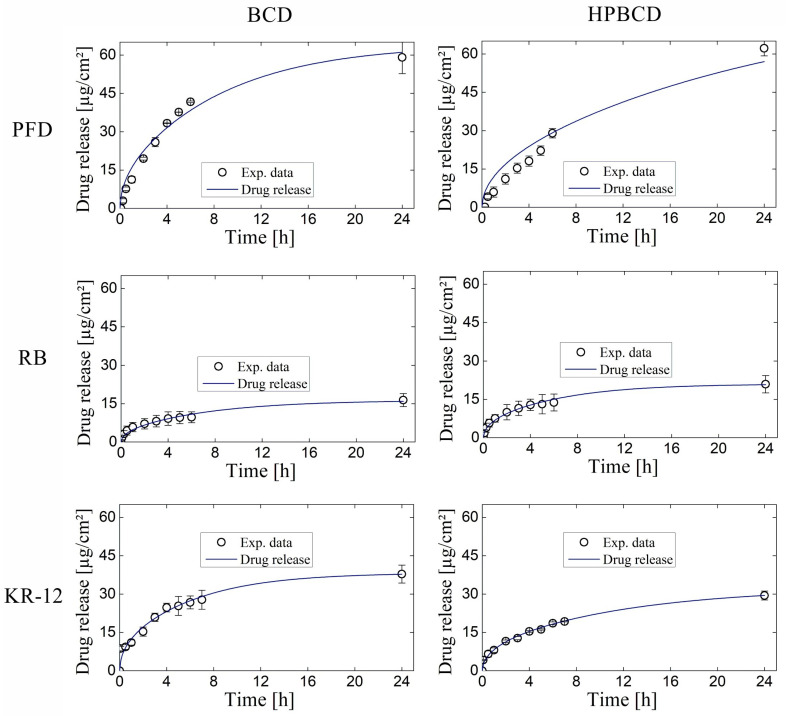
Drug release profiles simulated by COMSOL Multiphysics for experiments using smooth implants.

**Figure 10:**
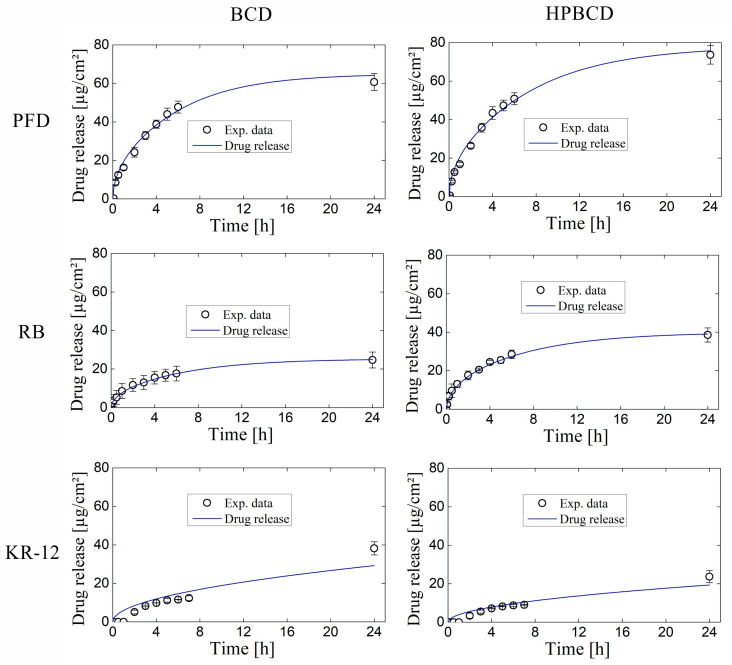
Drug release profiles simulated by COMSOL Multiphysics for experiments using textured implants.

Finally, Figure 9 and Figure 10 depict drug release profiles simulated by COMSOL Multiphysics for experiments involving smooth implants and textured samples, respectively. The results are aligned with the experimental data for both BCD and HPBCD coatings. The drug release simulations conducted with COMSOL Multiphysics were also consistent with the profiles generated by the mathematical model (Figure 5 and Figure 6), thereby confirming the effectiveness of the proposed unidirectional diffusion mathematical model. Interestingly, COMSOL Multiphysics simulations for the release of KR-12 from textured samples coated with both BCD and HPBCD polymers did not align completely with the experimental data, especially in the initial points where a lag time is observed. This discrepancy between experimental data and simulated profiles is also evident in the mathematical modeling, suggesting the involvement of other phenomena in the process. Given that KR-12 is a large molecule with multiple functional groups, interactions with the three-dimensional polymer matrix could be more pronounced through the thick coatings of T-BCD and T-HPBCD.

## 5. Conclusions

Drug release profiles were determined from commercial breast implants with both smooth and textured surfaces, following their functionalization with BCD and HPBCD polymers. The model drugs employed in these studies were PFD (Mw = 185.22 g/mol), RB (Mw = 973.67 g/mol), and KR-12 (Mw = 1570.93 g/mol). A mathematical model was applied to simulate drug release profiles and diffusion through the polymers. This model adhered to a unidirectional diffusion process in accordance with Fick’s second law and was numerically resolved via the orthogonal collocation method. The results established that the Sh number was sufficiently high to affirm that diffusion governed the processes under consideration. Notably, for the case of functionalized smooth samples, with their relatively thin polymer coatings (measuring between 14 and 20 μm), the diffusivity values ranged from 0.35 to 1 μm^2^/h, with no discernible correlation to the molecular weight of the drugs. However, for the functionalized textured samples, characterized by their thicker and irregular coatings (approximately 100 μm), their diffusivity values fell within the range of 0.1 to 6.5 μm^2^/h. In this case, the results indicated that mass diffusion was influenced by the molecular weight of the released drugs. Moreover, values for β were calculated, and they exhibited a direct dependence on the molecular weights of the drugs. These findings suggest that at higher β values, a greater amount of drug was retained within the polymer matrix upon reaching equilibrium. Finally, by integrating *D* and β parameters into COMSOL Multiphysics simulations, the validity of the unidirectional diffusion mathematical model was confirmed.

While the drug release profile simulations were consistent for PFD and RB molecules, discrepancies were observed for KR-12, possibly due to physisorption interactions between the peptide and samples. Therefore, it is recommended to explore other models that consider this phenomenon. Additionally, experimental diffusion profiles are crucial and should be investigated in further studies. Techniques such as fluorescent tracking could be employed to validate the obtained theoretical results. Another recommendation for future research is to study the thickness of cyclodextrin polymers on the implants, as this is a key parameter for controlling the release.

## Figures and Tables

**Table 1:** Drug release experiments categorized by breast implant type, cyclodextrin polymer coating, and released drug.

Breast Implant	Cyclodextrin Polymer	Drug	Label
Smooth	β-cyclodextrin	Pirfenidone	S-BCD/PFD
		Rose Bengal	S-BCD/RB
		KR-12	S-BCD/KR-12
	(2-hydroxypropyl)-β-cyclodextrin	Pirfenidone	S-HPBCD/PFD
		Rose Bengal	S-HPBCD/RB
		KR-12	S-HPBCD/KR-12
Textured	β-cyclodextrin	Pirfenidone	T-BCD/PFD
		Rose Bengal	T-BCD/RB
		KR-12	T-BCD/KR-12
	(2-hydroxypropyl)-β-cyclodextrin	Pirfenidone	T-HPBCD/PFD
		Rose Bengal	T-HPBCD/RB
		KR-12	T-HPBCD/KR-12

## Data Availability

The data that support the findings of this study are available from the corresponding author upon reasonable request.

## Appendix A. Collocation Method for the Numerical Solution

The collocation method [37] proposes to assume that the concentration, $C\left(x,t\right)$, has the next polynomial structure:

(A1) $$C\left(x,t\right)=\sum_{i=0}^{n}\phi_{i}\left(t\right)x^{i}=p\left(x\right)\Phi \left(t\right)$$

where the polynomial coefficients $\phi_{i}$, i=0,1,2,…,n, are time dependent. Here, $p\left(x\right)=\left(\begin{array}{ccccc} 1 & x & x^{2} & \cdots & x^{n} \end{array}\right)$ and

$$\Phi \left(t\right)=\left(\begin{array}{c} \phi_{0}\left(t\right)\\ \phi_{1}\left(t\right)\\ \phi_{2}\left(t\right)\\ \vdots \\ \phi_{n}\left(t\right) \end{array}\right).$$

Thus, the first and second partial derivative with respect to position, *x*, are

$$\begin{array}{ccc} \frac{\partial C\left(x,t\right)}{\partial x} & = & \sum_{i=0}^{n}i\phi_{i}\left(t\right)x^{i-1}=q\left(x\right)\Phi \left(t\right)\\ \frac{\partial^{2}C\left(x,t\right)}{\partial x^{2}} & = & \sum_{i=0}^{n}i\left(i-1\right)\phi_{i}\left(t\right)x^{i-2}=r\left(x\right)\Phi \left(t\right) \end{array}$$

where $q\left(x\right)=\left(\begin{array}{ccccc} 0 & 1 & 2x & \cdots & nx^{n-1} \end{array}\right)$ and $r\left(x\right)=\left(\begin{array}{ccccc} 0 & 0 & 2 & \cdots & n\left(n-1\right)x^{n-2} \end{array}\right).$

The problem reduces to find the vector of functions $\Phi \left(t\right)$. To achieve this goal, the following collocation points are defined: $0=x_{0}<x_{1}<x_{2}<\cdots <x_{n-1}<x_{n}=L$ in order to find the concentrations at these points:

$$\hat{C}\left(t\right)=\left(\begin{array}{c} C\left(x_{0},t\right)\\ C\left(x_{1},t\right)\\ \vdots \\ C\left(x_{n-1},t\right)\\ C\left(x_{n},t\right) \end{array}\right);$$

according to Equation (A1) it holds that

(A2) $$\hat{C}\left(t\right)=P\Phi \left(t\right)$$

where

$$P=\left(\begin{array}{ccccc} 1 & x_{0} & x_{0}^{2} & \cdots & x_{0}^{n}\\ 1 & x_{1} & x_{1}^{2} & \cdots & x_{1}^{n}\\ \vdots & \vdots & \vdots & \ddots & \vdots \\ 1 & x_{n} & x_{n}^{2} & \cdots & x_{n}^{n} \end{array}\right),$$

while the first and second partial derivatives derivative with respect to the position are:

$$\begin{array}{ccc} \frac{\partial \hat{C}\left(t\right)}{\partial x} & = & Q\Phi \left(t\right)\\ \frac{\partial^{2}\hat{C}\left(t\right)}{\partial x^{2}} & = & R\Phi \left(t\right) \end{array}$$

where

$$Q=\left(\begin{array}{cccccc} 0 & 1 & 2x_{0} & 3x_{0}^{2} & \cdots & nx_{0}^{n-1}\\ 0 & 1 & 2x_{1} & 3x_{1}^{2} & \cdots & nx_{1}^{n-1}\\ \vdots & \vdots & \vdots & \vdots & \ddots & \vdots \\ 0 & 1 & 2x_{n} & 3x_{n}^{2} & \cdots & nx_{n}^{n-1} \end{array}\right)$$

and

$$R=\left(\begin{array}{ccccccc} 0 & 0 & 2 & 6x_{0} & 12x_{0}^{2} & \cdots & n\left(n-1\right)x_{0}^{n-2}\\ 0 & 0 & 2 & 6x_{1} & 12x_{1}^{2} & \cdots & n\left(n-1\right)x_{1}^{n-2}\\ \vdots & \vdots & \vdots & \vdots & \vdots & \ddots & \vdots \\ 0 & 0 & 2 & 6x_{n} & 12x_{n}^{2} & \cdots & n\left(n-1\right)x_{n}^{n-2} \end{array}\right).$$

From Equation (A2), it is possible to compute the vector of functions as $\Phi \left(t\right)=P^{-1}\hat{C}\left(t\right)$, thus, in the collocations points, the partial derivatives with respect to position become

(A3) $$\begin{array}{ccc} \frac{\partial \hat{C}\left(t\right)}{\partial x} & = & M_{1}\hat{C}\left(t\right) \end{array}$$

(A4) $$\begin{array}{ccc} \frac{\partial^{2}\hat{C}\left(t\right)}{\partial x^{2}} & = & M_{2}\hat{C}\left(t\right) \end{array}$$

where $M_{1}=QP^{-1}$ and $M_{2}=RP^{-1}$. Finally, taking into account the approximation given in Equation (A2), the integral of the concentration along position must be

$$\int C\left(x,t\right)dx=\sum_{i=0}^{n}\phi_{i}\left(t\right)\frac{x^{i+1}}{i+1}=\left(\begin{array}{ccccc} x & \frac{x^{2}}{2} & \frac{x^{3}}{3} & \cdots & \frac{x^{n+1}}{n+1} \end{array}\right)\left(\begin{array}{c} \phi_{0}\left(t\right)\\ \phi_{1}\left(t\right)\\ \phi_{2}\left(t\right)\\ \vdots \\ \phi_{n}\left(t\right) \end{array}\right).$$

Therefore, the integral of the concentration in the polymer matrix is

$$\frac{1}{L}\int_{0}^{L}C\left(x,t\right)dx=\left(\begin{array}{ccccc} 1 & \frac{L}{2} & \frac{L^{2}}{3} & \cdots & \frac{L^{n}}{n+1} \end{array}\right)\left(\begin{array}{c} \phi_{0}\left(t\right)\\ \phi_{1}\left(t\right)\\ \phi_{2}\left(t\right)\\ \vdots \\ \phi_{n}\left(t\right) \end{array}\right)=I\Phi \left(t\right)$$

where $I=\left(\begin{array}{ccccc} 1 & \frac{L}{2} & \frac{L^{2}}{3} & \cdots & \frac{L^{n}}{n+1} \end{array}\right)$. As a function of $\hat{C}\left(t\right)$, this integral becomes

(A5) $$\frac{1}{L}\int_{0}^{L}C\left(x,t\right)dx=M_{3}\hat{C}\left(t\right)$$

where $M_{3}=IP^{-1}$.

The model given by the partial differential Equation (1), the initial condition (2), and the boundary conditions (3) and (8) at the collocation points is

(A6) $$\frac{\partial C\left(x_{i},t\right)}{\partial t}=D\frac{\partial^{2}C\left(x_{i},t\right)}{\partial x^{2}},\;t>0,\;i=1,2,\ldots ,n-1.$$

(A7) $$C\left(x_{i},0\right)=C_{0}\left(x_{i}\right),\;i=1,2,\ldots ,n-1.$$

(A8) $$\begin{array}{ccc} {\left[\frac{\partial C}{\partial x}\right]}_{x=x_{0}} & = & 0 \end{array}$$

(A9) $$\begin{array}{ccc} C\left(x_{n},t\right) & = & \gamma C_{l}\left(t\right)=\beta \left[\frac{\int_{0}^{L}C_{0}\left(x\right)dx}{L}-M_{3}\hat{C}\left(t\right)\right] \end{array}$$

By defining the vector

$$H_{0}=\left(\begin{array}{ccccc} 1 & 0 & \cdots & 0 & 0 \end{array}\right),\;y\;H_{n}=\left(\begin{array}{ccccc} 0 & 0 & \cdots & 0 & 1 \end{array}\right),$$

boundary conditions can be represented as

(A10) $$H_{0}M_{1}\hat{C}\left(t\right)=0$$

and

(A11) $$\left(H_{n}+\beta M_{3}\right)\hat{C}\left(t\right)=\beta M_{3}{\hat{C}}_{0},$$

respectively, where ${\hat{C}}_{0}=\hat{C}\left(0\right)$.

Finally, if the vector $\hat{C}\left(t\right)$ and matrices $H_{0}M_{1}$, $H_{n}+\beta M_{3}$, and $M_{2}$ are split as follows:

$$\hat{C}\left(t\right)=\left(\begin{array}{c} {\hat{C}}_{0}\left(t\right)\\ {\hat{C}}_{int}\left(t\right)\\ {\hat{C}}_{n}\left(t\right) \end{array}\right),$$

$$H_{0}M_{1}=\left(\begin{array}{ccc} {\left(H_{0}M_{1}\right)}_{0} & {\left(H_{0}M_{1}\right)}_{int} & {\left(H_{0}M_{1}\right)}_{n} \end{array}\right)$$

$$H_{n}+\beta M_{3}=\left(\begin{array}{ccc} {\left(H_{n}+\beta M_{3}\right)}_{0} & {\left(H_{n}+\beta M_{3}\right)}_{int} & {\left(H_{n}+\beta M_{3}\right)}_{n} \end{array}\right),$$

and

$$M_{2}=\left(\begin{array}{ccc} {\left(M_{2}\right)}_{0,0} & {\left(M_{2}\right)}_{0,int} & {\left(M_{2}\right)}_{0,n}\\ {\left(M_{2}\right)}_{int,0} & {\left(M_{2}\right)}_{int,int} & {\left(M_{2}\right)}_{int,n}\\ {\left(M_{2}\right)}_{n,0} & {\left(M_{2}\right)}_{n,int} & {\left(M_{2}\right)}_{n,n} \end{array}\right),$$

where ${\hat{C}}_{int}\left(t\right)$ describes the interior concentrations:

$${\hat{C}}_{int}\left(t\right)=\left(\begin{array}{c} C\left(x_{1},t\right)\\ C\left(x_{2},t\right)\\ \vdots \\ C\left(x_{n-1},t\right) \end{array}\right),$$

then the boundary conditions (A10) and (A11) can be used to compute the boundary concentrations based on interior concentrations as described in Equation (10), while considering the partial differential Equation (A6) for the interior nodes equations and the partial derivative (A4) the dynamical behavior of the interior concentrations is described by Equation (9).
